# Multi-pathological contributions toward atrophy patterns in the Alzheimer’s disease continuum

**DOI:** 10.3389/fnins.2024.1355695

**Published:** 2024-04-09

**Authors:** Rosaleena Mohanty, Daniel Ferreira, Eric Westman

**Affiliations:** ^1^Division of Clinical Geriatrics, Center for Alzheimer Research, Department of Neurobiology, Karolinska Institutet, Huddinge, Sweden; ^2^Facultad de Ciencias de la Salud, Universidad Fernando Pessoa Canarias, Las Palmas, Spain; ^3^Department of Neuroimaging, Center for Neuroimaging Sciences, Institute of Psychiatry, Psychology and Neuroscience, Kings College London, London, United Kingdom

**Keywords:** Alzheimer’s disease, atrophy patterns, multiple pathologies, amyloid-beta, tau, cerebrovascular burden

## Abstract

**Introduction:**

Heterogeneity in downstream atrophy in Alzheimer’s disease (AD) is predominantly investigated in relation to pathological hallmarks (Aβ, tau) and co-pathologies (cerebrovascular burden) independently. However, the proportional contribution of each pathology in determining atrophy pattern remains unclear. We assessed heterogeneity in atrophy using two recently conceptualized dimensions: *typicality* (typical AD atrophy at the center and deviant atypical atrophy on either extreme including limbic predominant to hippocampal sparing patterns) and *severity* (overall neurodegeneration spanning minimal atrophy to diffuse typical AD atrophy) in relation to Aβ, tau, and cerebrovascular burden.

**Methods:**

We included 149 Aβ + individuals on the AD continuum (cognitively normal, prodromal AD, AD dementia) and 163 Aβ− cognitively normal individuals from the ADNI. We modeled heterogeneity in MRI-based atrophy with continuous-scales of *typicality* (ratio of hippocampus to cortical volume) and *severity* (total gray matter volume). Partial correlation models investigated the association of typicality/severity with (a) Aβ (global Aβ PET centiloid), tau (global tau PET SUVR), cerebrovascular (total white matter hypointensity volume) burden (b) four cognitive domains (memory, executive function, language, visuospatial composites). Using multiple regression, we assessed the association of each pathological burden and typicality/severity with cognition.

**Results:**

(a) In the AD continuum, typicality (*r* = −0.31, *p* < 0.001) and severity (*r* = −0.37, *p* < 0.001) were associated with tau burden after controlling for Aβ, cerebrovascular burden and age. Findings imply greater tau pathology in limbic predominant atrophy and diffuse atrophy. (b) Typicality was associated with memory (*r* = 0.49, *p* < 0.001) and language scores (*r* = 0.19, *p* = 0.02). Severity was associated with memory (*r* = 0.26, *p* < 0.001), executive function (*r* = 0.24, *p* = 0.003) and language scores (*r* = 0.29, *p* < 0.001). Findings imply better cognitive performance in hippocampal sparing and minimal atrophy patterns. Beyond typicality/severity, tau burden but not Aβ and cerebrovascular burden explained cognition.

**Conclusion:**

In the AD continuum, atrophy-based severity was more strongly associated with tau burden than typicality after accounting for Aβ and cerebrovascular burden. Cognitive performance in memory, executive function and language domains was explained by typicality and/or severity and additionally tau pathology. Typicality and severity may differentially reflect burden arising from tau pathology but not Aβ or cerebrovascular pathologies which need to be accounted for when investigating AD heterogeneity.

## Introduction

Two entities that are increasingly recognized as critical players in Alzheimer’s disease (AD) are co-pathologies (Thal et al., 2014; DeTure and Dickson, 2019) and disease heterogeneity (Ferreira et al., 2020; Graff-Radford et al., 2021). Beyond the cardinal pathologies of amyloid-beta (Aβ) plaques and neurofibrillary tangles, the most frequent co-pathologies reported in AD are cerebrovascular disease, α-synuclein, and TDP-43 (Walker et al., 2015). Irrespective of whether AD heterogeneity manifests at the clinical syndromic level or biological level, differential involvement of co-pathologies has been reported (Murray et al., 2011; Ferreira et al., 2020; Graff-Radford et al., 2021; Jellinger, 2022; Polsinelli and Apostolova, 2022).

Studies investigating co-pathologies contributing to AD heterogeneity have had to rely on autopsy data as *in vivo* biomarkers are not readily accessible in most datasets for co-pathologies such as α-synuclein and TDP-43. However, the role of cerebrovascular co-pathology has remained poorly understood, despite available imaging markers. A possible reasoning could be that cerebrovascular pathology is very common in older age, and autopsy cases are end-stage and inevitably have some cerebrovascular burden, making it difficult to disentangle the role of this pathology (Toledo et al., 2013; Attems and Jellinger, 2014). Thus, to study cerebrovascular pathology, investigations with *in vivo* proxy markers at earlier disease stages may be required.

Recent neuroimaging studies have shown that treating disease heterogeneity as a continuous phenomenon may be more informative than categorizing individuals into subgroups or subtypes (Mohanty et al., 2022, 2023; Diaz-Galvan et al., 2023). In this approach, biological AD subtypes are defined by two dimensions, namely *typicality* and *severity* (Ferreira et al., 2020). As per this framework based on meta-analysis, typicality represents a spectrum which captures the involvement of the medial temporal regions relative to the neocortical regions. Along this dimension, typical AD atrophy lies in the middle and deviation from the middle represents limbic predominant atrophy on one extreme and hippocampal sparing atrophy on the other extreme. While involvement of both the medial temporal and neocortical regions represents typical AD atrophy in the middle of the typicality dimension, those individuals with relatively preserved medial temporal and neocortical regions may also lie in the middle. Complementing typicality, the second dimension of severity comes into play here, which distinguishes individuals with diffuse atrophy from those lacking overt atrophy. Every individual can be represented as a combination of the typicality and severity dimensions. Where an individual lies relative to the two dimensions is driven by their risk, protective and pathological factors. Together, continuous scales of typicality and severity provide a framework to simultaneously consider the contributions of atypical biomarker profile and overall neurodegeneration. Several studies have characterized AD subtypes by looking at pathological burden based on Aβ, tau, and cerebrovascular factors independently (Whitwell et al., 2012; Risacher et al., 2017; Ferreira et al., 2018; Ten et al., 2018; Ossenkoppele et al., 2019; Vogel et al., 2021). However, the relative contribution of multiple pathologies toward AD heterogeneity remains unclear. Upstream pathologies (Aβ, tau, cerebrovascular burden, etc.) are known to differentially contribute to neurodegeneration in the brain (Ballatore et al., 2007; Zlokovic, 2011). Accounting for heterogeneity in neurodegeneration, whether specific pathologies better explain cognitive performance also remains to be characterized.

Given that atrophy is downstream to several pathologies, we investigated AD heterogeneity in atrophy by the dimensions of typicality and severity in the AD continuum. We characterized how these two dimensions of heterogeneity (a) are associated with pathological burden assessed *in vivo* (Aβ, tau, cerebrovascular), and (b) explain cognitive abilities upon accounting for pathological burden.

## Methods

### Participants

We selected participants from the Alzheimer’s disease neuroimaging initiative (ADNI; launched in 2003; PI: Michael W. Weiner; http://adni.loni.usc.edu/), which aims to assess the progression of prodromal and early AD using biomarkers, clinical and neuropsychological assessments. We selected 312 individuals including 149 Aβ+ individuals (81 cognitively normal, 43 prodromal AD, 25 AD dementia), and 163 Aβ− cognitively normal individuals. Individuals were selected from ADNI 2–3 by including the first available visit with tau PET and concurrent Aβ PET and MRI scans. Aβ status was determined through Aβ PET (florbetapir standardized uptake value ratio or SUVR cutpoint = 1.11 (Joshi et al., 2012); or florbetaben SUVR cutpoint = 1.08 from http://adni.loni.usc.edu/). Detailed inclusion and exclusion criteria for the ADNI can be found at http://adni.loni.usc.edu/methods/. All procedures performed in the ADNI involving human participants were in accordance with the ethical standards of the local institutional review boards and with the 1964 Helsinki declaration and its later amendments. Informed consent was obtained from all participants included in the study.

### Neuroimaging

We included cross-sectional and concurrent Aβ PET to assess Aβ burden, tau PET to assess tau burden and MRI to assess atrophy and white matter hypointensity volume as a proxy for cerebrovascular burden. All scans per individual were acquired within 90 days of each other.

Aβ PET data were collected during a 50–70 min interval following a 370 MBq bolus injection of 18F-Florbetapir or during a 90 min following a 300 MBq ± 20% of 18F-florbetaben. Scans for both tracers were acquired in 4 × 5 min frames. Scans were motion corrected and averaged to obtain a mean image.^1^ Standard FreeSurfer parcellation of the MRI (Desikan et al., 2006) was applied to the co-registered Aβ PET images to extract global Aβ standardized uptake value ratio (SUVR) values intensity normalized to the whole cerebellum for the two tracers (Landau et al., 2015).^2^^,^^3^ The global Aβ SUVR was computed over cortical regions including frontal, anterior/posterior cingulate, lateral parietal, and lateral temporal regions. For comparison across the two tracers, SUVR were converted to centiloid scale for each tracer based on previously established equations (Royse et al., 2021).

Tau PET were collected by injecting 18F AV-1451 with a dosage of 370 MBq (10.0 mCi) ± 10% and scans were acquired between 75 and105 min post-injection. Dynamic acquisition was 30 min long with 6 × 5 min frames. Tau-PET scans were processed using the PetSurfer Toolbox (Greve et al., 2016) within FreeSurfer 6.0.0. Partial volume correction was conducted using the symmetric geometric matrix method (Rousset et al., 1998). Regional values were quantified in terms of the SUVR for the standard FreeSurfer parcellation of the MRI (Desikan et al., 2006) and averaged across all cortical and subcortical regions except the hippocampus (Ikonomovic et al., 2016; Lowe et al., 2016) to obtain global tau PET SUVR, computed with the cerebellum gray matter as the reference.

MRI were collected on 3.0 T scanners with 3-D accelerated T1-weighted sequences acquired sagittally with voxel size 1.1 × 1.1 × 1.2 mm^3^ (detailed protocol can be found at: http://adni.loni.usc.edu/methods/mri-tool/mri-analysis/). The MRI data were processed through TheHiveDB system (Muehlboeck et al., 2014) using FreeSurfer 6.0.0.^4^ Resulting segmentations were visually screened for quality control (1 case was excluded due to major overestimation of intracranial volume). Automatic region of interest segmentation yielded volumes in cortical and subcortical structures (Fischl et al., 2002; Desikan et al., 2006) representing brain atrophy. We used values of white matter hypointensity volume detected by FreeSurfer based on the automatic labeling using a probabilistic procedure (Fischl et al., 2002). Although cerebrovascular disease may manifest in the form of microbleeds, lacunes, enlarged perivascular spaces, etc. (Wardlaw et al., 2013; Duering et al., 2023), here we used readily accessible white matter hypointensity volume from FreeSurfer as a proxy for pathological burden with presumed vascular origin. It has previously been demonstrated that white matter hypointensity volume can reflect integrity of cerebrovascular health in aging and the AD continuum (Cedres et al., 2020; Nemy et al., 2020; Cedres et al., 2022; Zapater-Fajarí et al., 2023). All primary analyses were conducted using white matter hypointensity volumes based on T1-weighted MRI only. White matter hyperintensity volumes are more conventionally used to represent cerebrovascular burden. Thus, we also conducted supplementary analyses using white matter hyperintensity (based on T1-weighted and FLAIR sequences) in place of white matter hypointensity (Supplementary material S1). All volume measures were normalized by the estimated intracranial volume based on a residual approach (Voevodskaya et al., 2014).

### Atrophy-based patterns

We modeled heterogeneity in atrophy as a continuous phenomenon as demonstrated in prior studies (Mohanty et al., 2022, 2023; Diaz-Galvan et al., 2023). To this end, we quantified the atrophy-based patterns with two measures on a continuous scale. *Typicality* was proxied by the ratio of hippocampal volume to cortical volume to capture the atypical patterns (lower values on this scale indicate limbic predominance). *Severity* was proxied by the total gray matter volume adjusted for estimated intracranial volume to capture the overall disease burden or neurodegeneration (lower values on this scale indicate higher severity).

### Cognitive performance

Global cognition was assessed with the mini-mental state examination (MMSE). Additionally, we included composite scores assessing four cognitive domains including memory, executive function, language, and visual–spatial abilities provided by the phenotype harmonization consortium (Mukherjee et al., 2023).

### Statistical analysis

We analyzed the relationship between atrophy-based typicality and severity dimensions with a linear regression model. We correlated typicality and severity with age, sex (men vs. women) and *APOE ε4* carriership (carriers vs. non-carriers) using linear partial correlation models (Pearson’s and Spearman’s models were used for continuous and categorical variables respectively). Addressing the first aim of the study of how typicality and severity relate to pathological burden, we analyzed the association of each of the two dimensions with global Aβ PET SUVR, global tau PET SUVR, and white matter hypointensity volume using Pearson’s linear partial correlation models while controlling for age. Addressing the second aim of the study of how typicality and severity relate to cognitive performance, we analyzed the association of each dimension with composite scores for the cognitive domains of memory, executive function, language, and visual–spatial abilities using Pearson’s linear partial correlation models while controlling for pathological burden (global Aβ PET SUVR, global tau PET SUVR, white matter hypointensity volume). In all linear partial correlation models, we controlled for severity when examining typicality and vice versa, to evaluate whether the associations may be solely explainable by one dimension. To understand the relative contribution of different pathologies, we conducted multiple regression analyses following up only the above associations which were significant (*p* < 0.05). The cognitive performance was treated as the dependent variable and global Aβ PET SUVR, global tau PET SUVR, white matter hypointensity volume were added as predictors in addition to typicality and severity. Age and education were included as potential covariates. We controlled for false discovery rate in multiple comparisons. All analyses were performed using R version 4.3.2.

## Results

### Participant characteristics

Comparing the AD continuum and Aβ− control group, we found significant differences in demographic, genetic, cognitive and biomarker status as expected (Table 1; Supplementary material S2). Supplementary analyses showed that white matter hyperintensity volumes and white matter hypointensity volumes were strongly correlated in this study sample (*r* = 0.93, *p* < 0.001).

**Table 1:** Participant characteristics.

	(Aβ+)			(Aβ−)	*p*-value
	Cognitively normal	Prodromal AD	AD dementia	Cognitively normal	
*N*	81	43	24	163	-
Age (years)	75.3 (7.1)	74.8 (7.6)	78.1 (8.4)	71.6 (5.9)	<0.001
Sex (% female)	47 (58)	21 (49)	12 (50)	97 (60)	0.33
Education (years)	16.7 (2.3)	15.7 (2.6)	15.8 (2.7)	16.9 (2.3)	0.019
*APOE ε4* carriers (%)	56	60	54	24	<0.001
MMSE	28.8 (1.5)	27.4 (2.4)	22.0 (4.3)	29.2 (1.0)	<0.001
Memory composite	0.8 (0.5)	0.1 (0.5)	−0.8 (0.6)	0.9 (0.5)	<0.001
Executive function composite	0.7 (0.5)	0.3 (0.5)	−0.3 (0.9)	0.9 (0.5)	<0.001
Language composite	0.7 (0.5)	0.4 (0.5)	−0.1 (0.8)	0.9 (0.5)	<0.001
Visuospatial composite	0.01 (0.4)	0.04 (0.3)	−0.4 (0.7)	0.1 (0.2)	0.011
Aβ PET SUVR (centiloid)	59.4 (31.6)	66.5 (27.5)	87.9 (40.9)	4.5 (7.5)	<0.001
Tau PET SUVR	1.5 (0.2)	1.6 (0.3)	2.1 (0.7)	1.4 (0.1)	<0.001
White matter Hypointensity (mm^3^)	4,507 (5,182)	6,605 (10,423)	6,215 (4,960)	2,840 (2,631)	<0.001

Kruskal-Wallis test was used to compare the continuous measures between Aβ+ and Aβ- groups. Chi-squared test was used to compare the categorical measures between Aβ+ and Aβ- groups. white matter hypointensity volume was adjusted for estimated intracranial volume.Aβ, amyloid-beta; AD, Alzheimer’s disease; N, sample size; APOE, Apolipoprotein E; MMSE, Mini Mental State Examination; SUVR, standardized uptake value.

### Association between atrophy-based typicality and severity

**Figure 1:**
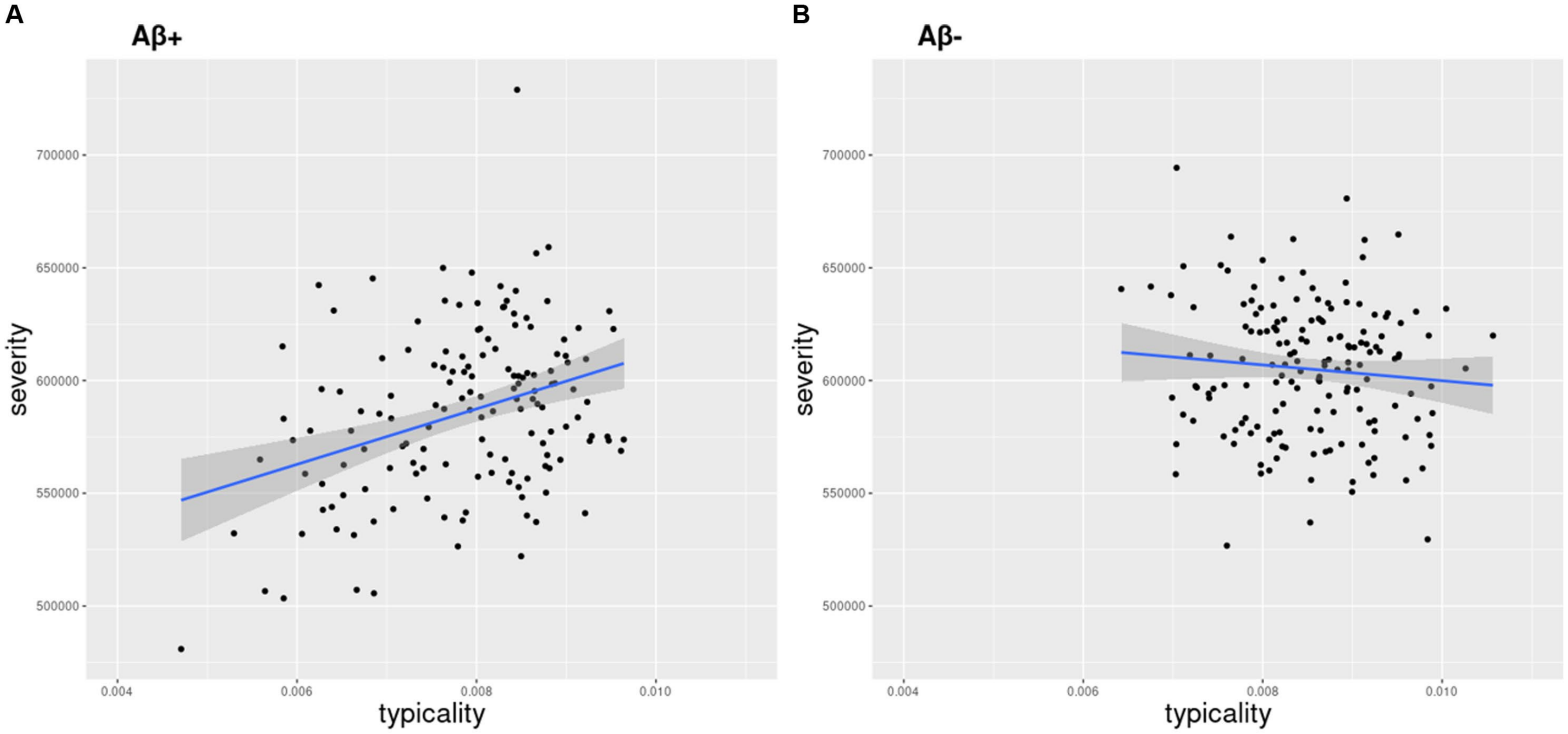
Relationship between atrophy-based typicality and severity. Typicality and severity were positively correlated in the **(A)** AD continuum (r = 0.34, *p* < 0.0001) but not in the **(B)** Aβ− control group (*r* = 0.09, *p* = 0.23). Typicality dimension (unitless) was quantified by the ratio of hippocampal volume to cortical volume. Lower values correspond to more limbic predominant atrophy. Severity dimension (expressed in mm^3^) was quantified by the total gray matter volume adjusted by estimated intracranial volume to capture the overall neurodegeneration. Lower values (volume) correspond to higher severity.

**Figure 2:**
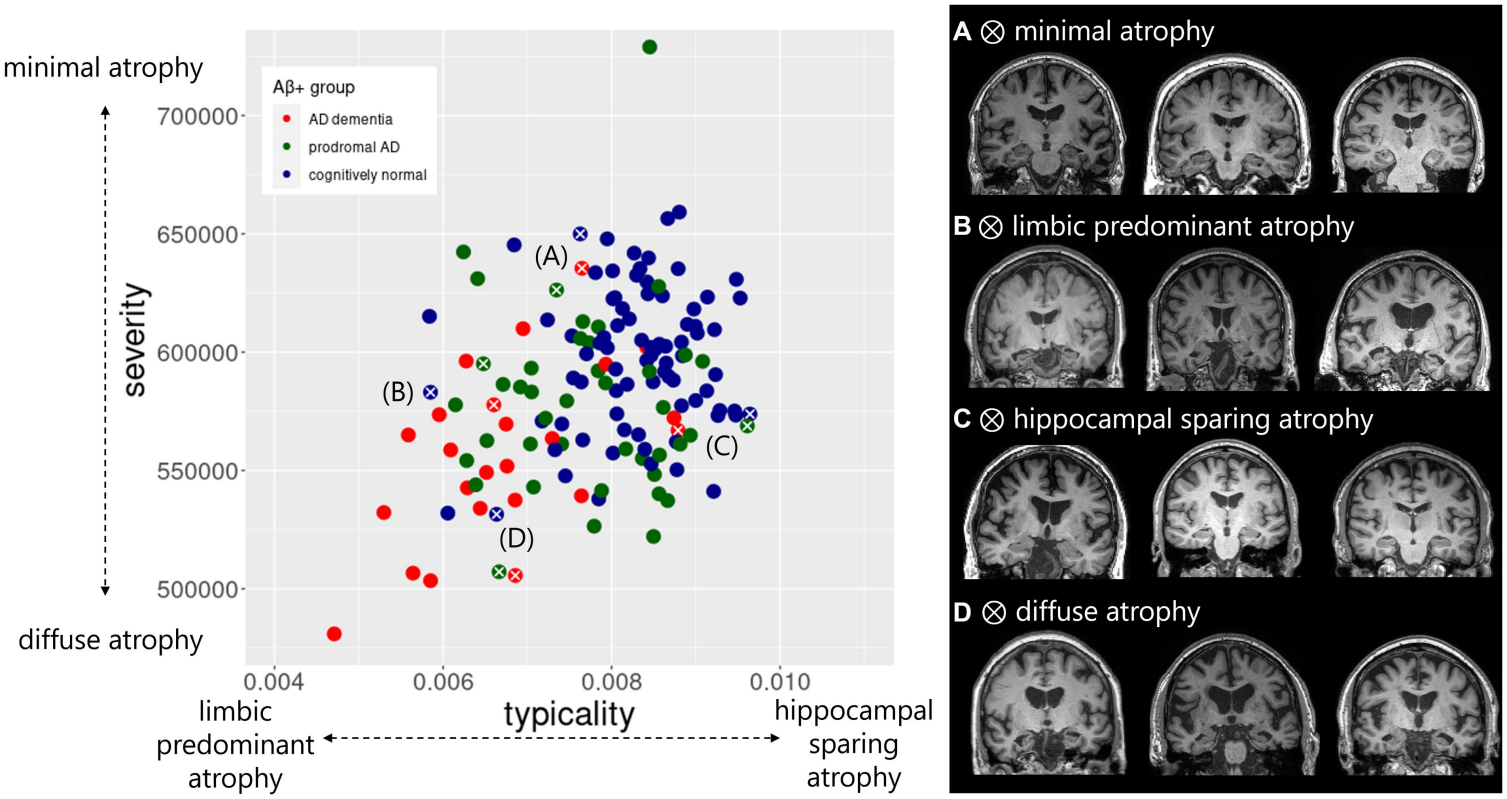
Atrophy patterns reflected by typicality and severity in the AD continuum. Distribution of typicality and severity are colored by clinical groups within AD continuum including AD dementia, prodromal AD, cognitively normal. Exemplar atrophy pattern based on MRI highlight the extreme cases along the two dimensions indicated by white cross correspond to **(A)** minimal atrophy, **(B)** limbic predominant atrophy, **(C)** hippocampal sparing atrophy, and **(D)** diffuse atrophy. For each of the four atrophy patterns, example brain images represent individuals with AD dementia (left column), prodromal AD (middle column) and cognitively normal status (right column). Typicality dimension (unitless) was quantified by the ratio of hippocampal volume to cortical volume. Severity dimension (expressed in mm^3^) was quantified by the total gray matter volume adjusted by estimated intracranial volume.

The distribution of typicality and severity measures highlighted a greater variance in the AD continuum compared to the Aβ− control group (Supplementary material S3). The AD continuum had significantly lower typicality (Kruskal-Wallis *H* = 27.6, *p* < 0.001) and higher severity (Kruskal-Wallis *H* = 23.2, *p* < 0.001) values compared to the Aβ − control group. Clinical groups within the AD continuum also differed pairwise in both typicality and severity dimensions (Supplementary material S3). The association between typicality and severity was significant in the AD continuum (Figure 1A, *R* = 0.34, *p* < 0.0001) but not in the Aβ − control group (Figure 1B, *R* = 0.09, *p* = 0.23). Within each clinical group of the AD continuum group, we found exemplars showing tendencies of four atrophy patterns (Figure 2). Along the extremes, the typicality dimension captured tendencies ranging from limbic predominant atrophy to hippocampal sparing atrophy whereas the severity dimension captured tendencies ranging from minimal atrophy to diffuse (typical AD) atrophy. The correlation suggests a closer association of limbic predominant atrophy and diffuse atrophy patterns on one end and that of hippocampal sparing atrophy and minimal atrophy patterns on the other end. Upon adjusting for clinical diagnosis (AD dementia, prodromal AD, cognitively normal) in the AD continuum, we observed that the association between typicality and severity was no longer significant (*r* = 0.08, *p* = 0.32).

### Characterization of atrophy-based typicality/severity by age, sex and *APOE ε4* carriership

In the AD continuum, the linear partial correlation model suggested that severity (*r_part_* = −0.38, *p* < 0.01) but not typicality (*r_part_* = −0.14, *p* = 0.10) was significantly associated with age. This finding suggests that less severe individuals tend to be younger while more severe individuals tend to be older, as expected. Neither of the dimensions showed significant association with sex (*p* > 0.1) or *APOE ε4* carriership (*p* > 0.07). In the Aβ− control group, linear partial correlation model suggested that both typicality (*r_part_* = −0.32, *p* < 0.001) and severity (*r_part_* = −0.49, *p* < 0.001) were significantly associated with age. Additionally, typicality (*r_part_* = 0.25, *p* = 0.001) but not severity (*r_part_* = −0.04, *p* = 0.56) was associated sex. Neither of the dimensions were associated with *APOE ε4* carriership (*p* > 0.47).

### Association between atrophy-based typicality/severity and pathological burden

Summarized in Table 2, we observed that both typicality and severity were significantly correlated with pathological burden (global Aβ SUVR, global tau SUVR, white matter hypointensity volume) in the AD continuum. Typicality was associated with global tau SUVR (*r_part_* = −0.31, *p* < 0.001) suggesting greater pathology in limbic predominant atrophy than hippocampal sparing. Relative to typicality, severity was more strongly associated with global tau SUVR (*r_part_* = −0.37, *p* < 0.001) suggesting greater pathology in diffuse atrophy than minimal atrophy. Neither typicality nor severity were associated with pathological burden in the Aβ− group. The significance of these results does not change upon substituting white matter hyperintensity volume in place of white matter hypointensity volume (Supplementary material S4).

**Table 2:** Relationship of atrophy-based typicality and severity with pathological burden.

Subtype dimension	Group	Global Aβ SUVR	Global tau SUVR	White matter hypointensity
Typicality	Aβ+	*r_part_* = 0.02	*r_part_* = −0.31	*r_part_* = −0.06
		*p* = 0.78	*p* < 0.001	*p* = 0.49
	Aβ−	*r_part_* = 0.10	*r_part_* = 0.04	*r_part_* = −0.03
		*p* = 0.20	*p* = 0.63	*p* = 0.72
Severity	Aβ+	*r_part_* = −0.04	*r_part_* = −0.37	*r_part_* = −0.03
		*p* = 0.61	*p* < 0.001	*p* = 0.68
	Aβ−	*r_part_* = 0.07	*r_part_* = −0.12	*r_part_* = 0.004
		*p* = 0.37	*p* = 0.14	*p* = 0.95

Linear partial correlation models examining relationship between typicality/severity with pathological burden. Model for each pathology was controlled for the other two pathologies and age. Additionally, models for typicality were controlled for severity and vice versa. White matter hypointensity was adjusted for estimated intracranial volume.Aβ, amyloid-beta; SUVR, Standardized Uptake Value Ratio; *r_part_*, linear partial correlation coefficient.

### Association between atrophy-based typicality/severity and cognitive performance

**Figure 3:**
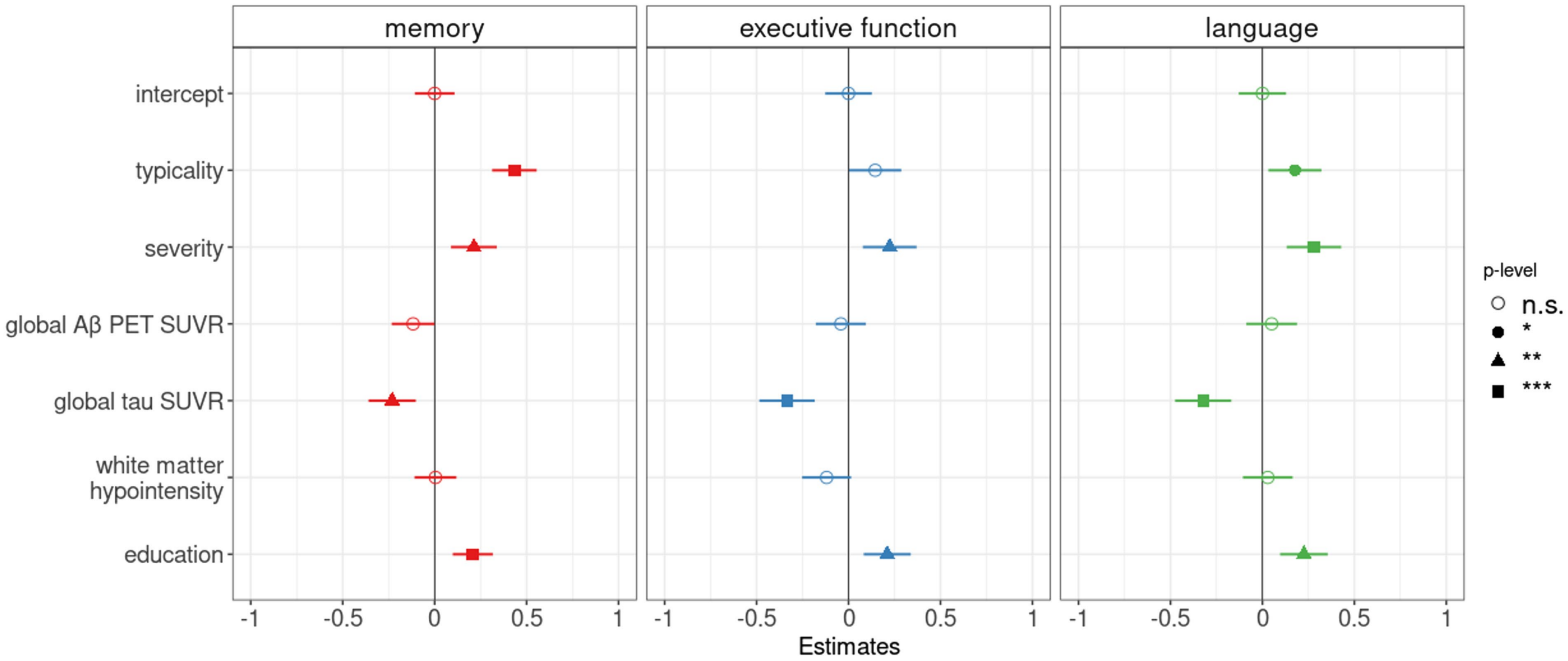
Pathological contributors and atrophy-based typicality and severity as correlates of cognitive performance in the AD continuum. Forest plot for multiple linear regression analyses showing the relative contribution of Aβ (global Aβ PET SUVR), tau (global tau PET SUVR) and cerebrovascular (estimated intracranial volume-adjusted white matter hypointensity) burden in addition to typicality and severity to explain composite memory, executive function, and language scores. As potential covariates, age and education were tested in all models. As age appeared to be a non-significant contributor in all models, the variable was dropped from the final models. The vertical line indicates no effect for each plot. Standardized point estimates and error bars are shown, significance levels were corrected for multiple comparisons and correspond to *p* < 0.05 (*), *p* < 0.01 (**), and *p* < 0.001 (***).

Summarized in Table 3 and Supplementary material S5, we observed that both typicality and severity were significantly associated with cognitive performance (memory, executive function, language domains) in the AD continuum after controlling for pathological burden (global Aβ SUVR, global tau SUVR, white matter hypointensity/hyperintensity volume). All correlations were positive indicating that hippocampal sparing atrophy had better cognitive scores than limbic predominant atrophy (along typicality), and minimal atrophy had better cognitive scores than diffuse atrophy (along severity). Following up on the above results for memory, executive function, language domains which were significant, we assessed the relative contribution of each pathology using multiple regression analyses as reported in Figure 3. With a variance inflation factor < 1.5 for all independent variables, we noted that multicollinearity was not an issue in the regression models. Typicality, severity and global tau SUVR were significantly associated with scores in the memory and language domains. Severity and global tau SUVR were significantly associated with executive function. Among the three pathological variables, global tau SUVR was the only significant explanatory variable of cognitive performance in all three domains. Among the covariates, age appeared to be a non-significant contributor in all models and was dropped from the final models. All significant results from multiple regression analyses remained unchanged when considering the contribution of white matter hyperintensity in place of white matter hypointensity (Supplementary material S6).

**Table 3:** Relationship of atrophy-based typicality and severity with cognitive performance.

Subtype dimension	Group	Memory	Executive function	Language	Visuospatial
Typicality	Aβ+	*r_part_* = 0.49	*r_part_* = 0.16	*r_part_* = 0.19	*r_part_* = −0.01
		*p* < 0.01	*p* = 0.053	*p* = 0.02	*p* = 0.90
	Aβ−	*r_part_* = 0.04	*r_part_* = −0.04	*r_part_* = −0.13	*r_part_* = 0.04
		*p* = 0.65	*p* = 0.63	*p* = 0.09	*p* = 0.75
Severity	Aβ+	*r_part_* = 0.26	*r_part_* = 0.24	*r_part_* = 0.29	*r_part_* = 0.11
		*p* < 0.01	*p* < 0.01	*p* < 0.01	*p* = 0.35
	Aβ−	*r_part_* = 0.02	*r_part_* = −0.06	*r_part_* = 0.04	*r_part_* = −0.17
		*p* = 0.81	*p* = 0.39	*p* = 0.57	*p* = 0.19

Linear partial correlation models examining relationship between typicality/severity with cognitive performance. All models were controlled for pathological burden (global Aβ SUVR, global tau SUVR, estimated intracranial volume adjusted white matter hypointensity volume). Models for typicality were controlled for severity and vice versa.Aβ, amyloid-beta; r_part_, linear partial correlation coefficient.

We conducted sensitivity analysis to examine potential outlier effect in the AD continuum (*n* = 1). Significant findings remained upon exclusion of the individual (Supplementary material S7).

## Discussion

In this study, we modeled heterogeneity in brain atrophy in relation to *in vivo* measures of Aβ, tau, and cerebrovascular burden in the AD continuum. Atrophy patterns were captured by the dimensions of typicality (degree of atypical atrophy) and severity (overall neurodegeneration). It is important to consider the contribution of both typicality and severity toward heterogeneity and this study shows that the dimensions were differentially associated with the three pathologies, and differentially explained cognitive performance upon accounting for pathological burden.

### Atrophy-based heterogeneity as a continuous phenomenon

Rather than categorizing individuals into groups to represent distinct atrophy patterns in most previous studies (Whitwell et al., 2012; Noh et al., 2014; Ferreira et al., 2017; Park et al., 2017; Risacher et al., 2017; Poulakis et al., 2018), we treated atrophy-based heterogeneity as a continuous phenomenon using the dimensions of typicality and severity (Ferreira et al., 2020). The continuous-scale approach has been shown to be more sensitive than the categorization approach in capturing heterogeneity in tau PET patterns in the AD continuum (Mohanty et al., 2023). Such an approach has been shown to be useful in tracking the differential response of atrophy-based subtypes to symptomatic treatment using donepezil in individuals with mild cognitive impairment (Diaz-Galvan et al., 2023). Further, the continuous-scale approach differentiated atrophy-based subtypes in individuals with autopsy-confirmed AD in susceptibility to co-pathologies including Lewy body and TDP-43 (Mohanty et al., 2022). The current study adds a multimodal perspective by showing that atrophy-based typicality and severity are differentially associated with *in vivo* Aβ, tau, and cerebrovascular burden and differentially explain cognitive performance in the AD continuum. Typicality and severity were correlates of age and sex in the Aβ− group only but were correlates of pathological burden and cognitive status in the AD continuum suggesting the potential of the two dimensions in capturing AD-related heterogeneity.

### Association between atrophy-based typicality and severity

Typicality and severity were conceptualized to determine the tendency of an individual to belong to one of the AD subtypes (Ferreira et al., 2020). The degree to which these two dimensions would capture characteristics of subtypes independently has been unclear so far. A previous operationalization of the framework found that typicality and severity dimensions were not significantly correlated when capturing heterogeneity in tau PET in the AD continuum (Mohanty et al., 2023). Using an overlapping sample (~85%) of the AD continuum, the two dimensions were significantly correlated when capturing heterogeneity in atrophy in the current study. These two studies may suggest that the continuous-scale characterization of heterogeneity does not directly translate between tau PET and MRI, probably because MRI-based atrophy is unspecific to AD and carries the impact of several pathologies beyond tau burden. This finding is complementary to and supported by a previous investigation which demonstrated that categorizing individuals into a subtype based on tau PET and MRI does not reflect the same subtype across the two modalities (Mohanty et al., 2020). Another study operationalizing atrophy-based subtypes using typicality and severity also did not yield a significant correlation between typicality and severity (Mohanty et al., 2022). The study, however, may have been limited by the small sample size of only autopsy-confirmed AD cases, thereby including end-stage cases (limited variability in severity by design). Typicality and severity may not be correlated when characterizing subtypes or patterns of atrophy in individuals with a comparable disease stage. On the contrary, the current study included the AD continuum (cognitively normal, prodromal AD, AD dementia), encompassing a wider spectrum of disease. When controlled for the clinical diagnosis, typicality and severity were no longer significant. This finding suggests that the relationship between atrophy-based typicality and severity in the AD continuum was driven by the later stages of the disease (AD dementia) aligning toward diffuse or limbic predominant atrophy patterns and pre-dementia cases aligning toward hippocampal sparing or minimal atrophy patterns. This finding is akin to the report suggesting that hippocampal sparing pattern in tau pathology may occur at earlier disease stages (Ferreira et al., 2022). Thus, the current study highlights that typicality and severity can be related when characterizing subtypes or patterns of atrophy in individuals across disease stages.

### Relationship between typicality/severity and pathological burden

Typicality and severity were differentially associated with *in vivo* measures of pathological (Aβ, tau, and cerebrovascular) burden. The typicality dimension was associated with global tau burden in the AD continuum. Based on this finding, limbic predominant atrophy may be associated with higher global tau burden. Limbic predominant atrophy has been shown to have higher tau burden, but only at a regional level in the entorhinal cortex but not at a global level (Ossenkoppele et al., 2019). Defined based on distribution of tau pathology (neurofibrillary tangle count), limbic predominant tau subtype has been reported to have lower tau burden than hippocampal sparing tau subtype (Murray et al., 2011; Janocko et al., 2012; Whitwell et al., 2012). Individuals with AD in these prior studies were at advanced Braak stages (V-VI). In contrast, the current study characterized heterogeneity based on atrophy in the AD continuum. An over-representation of predementia cases in the current cohort implies that many individuals are at earlier stages of tau spread (Table 1) considering a global tau PET SUVR >1.3 threshold (Maass et al., 2017). Thus, the tau burden observed in the current cohort may largely be driven by early Braak (transentorhinal-limbic) regions and may explain our finding of higher global tau burden in limbic predominant atrophy. Reconciling findings from prior and the current studies supports the notion that subtypes or patterns based on tau pathology and atrophy are not fully interchangeable especially in cohorts including pre-dementia stages (Mohanty et al., 2020, 2023). The severity dimension was a stronger correlate of global tau burden than typicality in the AD continuum. This result suggests that a diffuse atrophy pattern may be associated with higher tau burden, consistent with previous findings (Dong et al., 2017; Ossenkoppele et al., 2019). Collectively, these reports may indicate that despite representing distinct patterns, limbic predominant and diffuse atrophy patterns may possibly share a similar biological pathway, given their vulnerability toward AD and non-AD pathologies (Mohanty et al., 2022) and eventual convergence into widespread atrophy over time (Poulakis et al., 2022). Cumulative pathological accumulation may explain why some individuals with limbic predominant atrophy may evolve into a diffuse atrophy pattern when tracked longitudinally (Poulakis et al., 2022). We did not observe any association of typicality/severity with Aβ or cerebrovascular burden. Other studies have reported higher cerebrovascular burden (white matter hyperintensity, micro/macro infarcts, etc.) associated with limbic predominant atrophy based on autopsy validation in AD dementia (Whitwell et al., 2012), AD dementia in a memory clinic population (Ferreira et al., 2018), and prodromal AD (Ten et al., 2018).

### Relationship of typicality/severity and pathological burden with cognition

We observed that typicality and severity were correlated with cognitive performance in memory, executive function, and language domains in the AD continuum. Further, our findings delineated the relative contribution of Aβ, tau, and cerebrovascular burden toward cognition upon accounting for atrophy-based heterogeneity. The strongest pathological correlate was global tau burden across all three cognitive domains which is congruent with the evidence that tau pathology is closely related to cognitive symptoms in both typical and atypical AD (Ossenkoppele et al., 2016; Xia et al., 2017; Tetzloff et al., 2018). Global Aβ burden did not significantly contribute in explaining cognition, in line with studies which reported that Aβ pathology did not significantly differ when investigating heterogeneity in atrophy patterns in AD with different cognitive phenotypes (Risacher et al., 2017; Whitwell et al., 2018; Ferreira et al., 2020). White matter hypointensity also did not significantly explain cognitive performance possibly because severe cerebrovascular risks have been a part of exclusion criteria for ADNI. Altogether, atrophy-derived heterogeneity and tau pathology best explained cognitive variability in this cohort. The relative contribution of these pathologies in relation to typicality and severity should be further validated in other samples (e.g., clinical population) where vascular pathology is a very common finding.

It is important to consider the interpretation of the typicality and severity dimensions in this study. Lower and higher values of typicality correspond to relatively limbic predominant atrophy and hippocampal sparing atrophy, respectively. Similarly, lower (i.e., higher gray matter volumes) and higher (lower gray matter volumes) values of severity correspond to relatively minimal atrophy and diffuse atrophy, respectively. Such an interpretation is straightforward when examining heterogeneity in the AD dementia stage (Ferreira et al., 2020). However in the AD continuum, we observed a progressive shift in both typicality (AD dementia < prodromal AD < cognitively normal) and severity (AD dementia > prodromal AD > cognitively normal) in the current study. The progressive shift of typicality along the AD continuum should not be mis-interpreted as AD dementia always exhibiting limbic predominant atrophy or cognitively normal individuals always exhibiting hippocampal sparing atrophy. It is possible for cognitively normal individuals to have limbic predominant atrophy and AD dementia individuals to have hippocampal sparing atrophy (seen in Figure 2). Majority of the findings in this study support that severity is a relatively more consistent and stronger correlate of pathological burden and cognitive outcomes compared to typicality (in addition to being associated with typicality itself). This relative dominance of severity could be interpreted as cognitively normal individuals being less severe while AD dementia individuals being more severe, explaining the progressive shift of severity (and in turn of typicality) along the AD continuum. Thus, typicality and severity should be cautiously interpreted in the AD continuum.

This study has a few limitations. We approached the contribution of co-pathologies to atrophy-based patterns from the *in vivo* perspective by looking at cerebrovascular burden. White matter hypointensity/hyperintensity volume as a cerebrovascular marker is generally presumed to have vascular origin. It must however, be acknowledged that this marker is fairly unspecific to cerebrovascular disease due to its possible association with immune activation, blood brain barrier dysfunction, altered cell metabolic pathways, glial injury, etc. (Wardlaw et al., 2015). Predominance of white matter hyperintensity in posterior regions of the brain has particularly been related to cerebral amyloid angiopathy (Cozza et al., 2023). Use of total white matter hypointensity/hyperintensity volume lacks regional specificity and thus, it is unclear to what extent the marker captures cerebral amyloid angiopathy in the current study. We investigated the role of multiple pathologies by accounting for their global burden in the brain. Future studies should consider not only global but regional contributions of different pathologies toward heterogeneity in atrophy. Other commonly observed co-pathologies such as Lewy body and TDP-43 could not be accounted for due to lack of readily available biomarkers for these pathologies.

In conclusion, we operationalized heterogeneity in atrophy based on continuous measures of typicality and severity in the AD continuum. Adding perspectives from multiple pathologies, typicality and severity were associated with tau burden but not with Aβ or cerebrovascular burden. Typicality and severity along with tau burden also explained performance in memory, executive function, and language domains. Findings remain to be validated in real world population where comorbid pathologies may be relatively more prevalent. Altogether, we show that typicality and severity can reflect complementary multi-pathological contributions to better understand AD heterogeneity.

## Data availability statement

Publicly available datasets were analyzed in this study. This data can be found at: https://adni.loni.usc.edu/.

## Ethics statement

The studies involving humans were approved by Local institutional review boards (IRB) for each center within ADNI (Albany Medical Center Committee on Research Involving Human Subjects IRB, Boston University Medical Campus and Boston Medical Center IRB, Butler Hospital IRB, Cleveland Clinic IRB, Columbia University Medical Center IRB, Duke University Health System IRB, Emory IRB, Georgetown University IRB, Health Sciences IRB, Houston Methodist IRB, Howard University Office of Regulatory Research Compliance, Icahn School of Medicine at Mount Sinai Program for the Protection of Human Subjects, Indiana University IRB, IRB of Baylor College of Medicine, Jewish General Hospital Research Ethics Board, Johns Hopkins Medicine Institutional Review Board, Lifespan—Rhode Island Hospital IRB, Mayo Clinic IRB, Mount Sinai Medical Center IRB, Nathan Kline Institute for Psychiatric Research & Rockland Psychiatric Center Institutional Review Board, New York University Langone Medical Center School of Medicine Institutional Review Board, Northwestern University IRB, Oregon Health and Science University IRB, Partners Human Research Committee Research Ethics, Board Sunnybrook Health Sciences Centre, Roper St. Francis Healthcare Institutional Review Board, Rush University Medical Center IRB, St. Joseph’s Phoenix IRB, Stanford IRB, The Ohio State University IRB, University Hospitals Cleveland Medical Center Institutional Review Board, University of Alabama Office of the IRB, University of British Columbia Research Ethics Board, University of California Davis IRB Administration, University of California Los Angeles Office of the Human Research Protection Program, University of California San Diego Human Research Protections Program, University of California San Francisco Human Research Protection Program, University of Iowa Institutional Review Board, University of Kansas Medical Center Human Subjects Committee, University of Kentucky Medical IRB, University of Michigan Medical School IRB, University of Pennsylvania IRB, University of Pittsburgh IRB, University of Rochester Research Subjects Review Board, University of South Florida IRB, University of Southern, California IRB, UT Southwestern IRB, VA Long Beach Healthcare System IRB, Vanderbilt University Medical Center IRB, Wake Forest School of Medicine IRB, Washington University School of Medicine IRB, Western IRB, Western University Health Sciences Research Ethics Board, and Yale University IRB). The studies were conducted in accordance with the local legislation and institutional requirements. The participants provided their written informed consent to participate in this study.

## Author contributions

RM: Conceptualization, Data curation, Formal analysis, Funding acquisition, Investigation, Methodology, Project administration, Resources, Software, Supervision, Validation, Visualization, Writing – original draft, Writing – review & editing. DF: Conceptualization, Investigation, Methodology, Supervision, Writing – review & editing, Funding acquisition, Project administration, Resources, Data curation, Formal analysis, Software, Validation, Visualization. EW: Conceptualization, Investigation, Methodology, Supervision, Writing – review & editing, Funding acquisition, Project administration, Resources, Data curation, Formal analysis, Software, Validation, Visualization.

## Footnotes

^1^ http://adni.loni.usc.edu/methods/pet-analysis-method/pet-analysis/

^2^ https://adni.bitbucket.io/reference/docs/UCBERKELEYAV45/ADNI_AV45_Methods_JagustLab_06.25.15.pdf

^3^ https://adni.bitbucket.io/reference/docs/UCBERKELEYFBB/UCBerkeley_FBB_Methods_04.11.19.pdf

^4^ http://freesurfer.net/
