# Supplementary material for: Multi-pathological contributions toward atrophy patterns in the Alzheimer’s disease continuum
Source: Front Neurosci. 2024 Apr 9;18:1355695. doi: 10.3389/fnins.2024.1355695 (PMC11036869; doi:10.3389/fnins.2024.1355695)
Supplement: Supplementary file 1 [file Data_Sheet_1.pdf]

Multi-pathological contributions towards atrophy patterns in the Alzheimer's disease continuum

Supplementary Material

S1. Comparison of white matter hyperintensity versus hypointensity volume

We extracted white matter hyperintensity volume measures which are made available by ADNI. White matter hyperintensity measurement was based on a Bayesian approach (DeCarli et al. 1999) for segmentation of high resolution 3D T1 and FLAIR sequences. White matter hyperintensity volumes were adjusted for estimated intracranial volume using the residual approach (Voevodskaya et al. 2014). All the main analyses involving white matter hypointensity volume were conducted using white matter hypointensity volume instead. Results from supplementary analyses by replacing white matter hyperintensity instead of white matter hypointensity are reported in **S4-S6**.

S2. Distribution of tau PET composite SUVR across clinical groups in the study sample

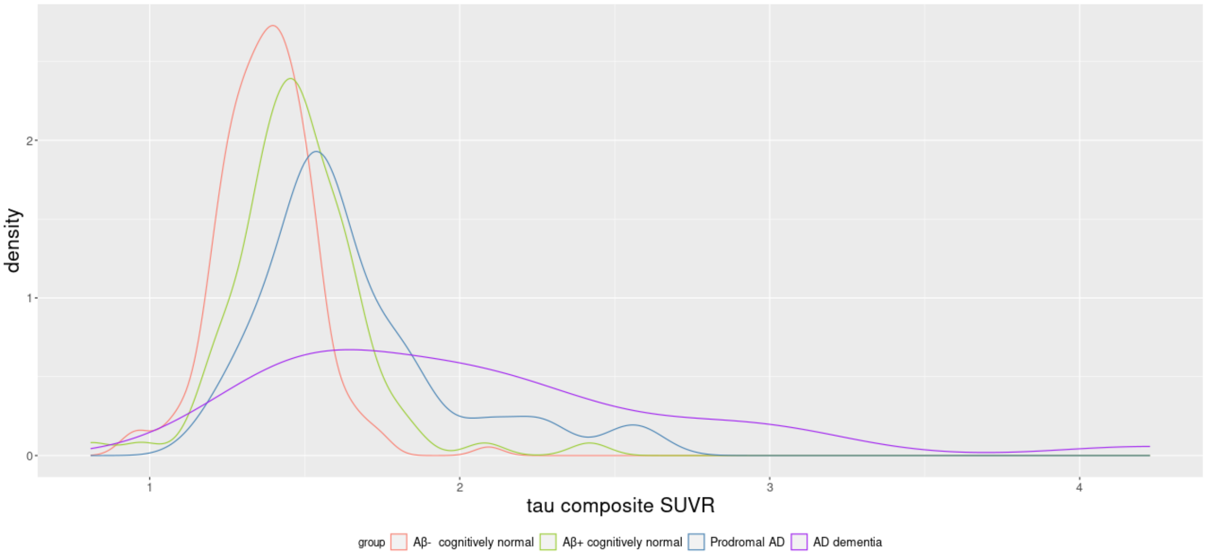

There was a significant difference in tau composite (global) SUVR among the four groups (**Table 1** in the main text). Post-hoc pairwise comparisons showed that each of the AD dementia (Kruskal-Wallis H = 40.3, p<0.001), prodromal AD (Kruskal-Wallis H = 40.6, p<0.001) and Aβ+ cognitively normal (Kruskal-Wallis H = 18.1, p<0.001) groups had significantly higher tau PET SUVR than the Aβ- cognitively normal control group.

S3. Distribution of atrophy-based (A) typicality and (B) severity measures in the study sample

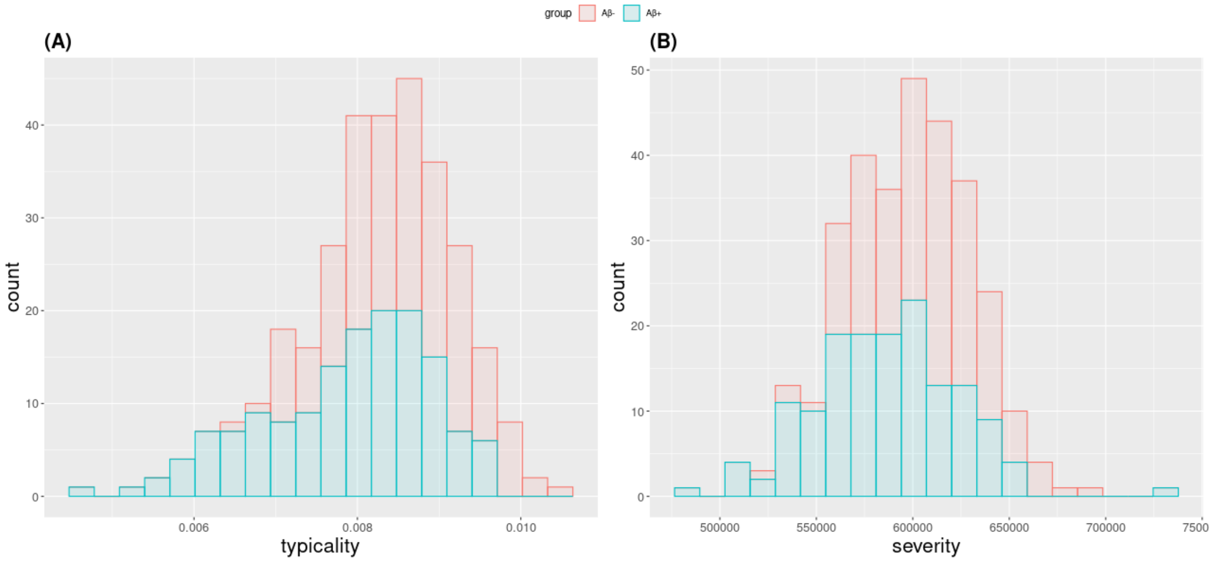

Typicality dimension was quantified by the ratio of hippocampal volume to cortical volume (unitless). Lower values correspond to more limbic predominant atrophy. Severity dimension was quantified by the total gray matter volume adjusted by estimated intracranial volume (mm^3) to capture the overall neurodegeneration. Lower values (volume) correspond to higher severity. Both typicality (Kruskal-Wallis H = 27.6, p<0.001) and severity (Kruskal-Wallis H = 23.2, p<0.001) were significantly different in the AD continuum (Aβ+) compared to the Aβ− control group. Within the AD continuum, Dunn's post-hoc test showed significant pairwise differences in typicality: AD dementia < prodromal AD (Z=-2.9, p=0.003), AD dementia < cognitively normal (Z=-5.8, p<0.001), prodromal AD < cognitively normal (Z=-3.1, p=0.002). Similarly, there were significant pairwise differences in severity: AD dementia < prodromal AD (Z=-1.9, p=0.04), AD dementia < cognitively normal (Z=-4.6, p<0.001), prodromal AD < cognitively normal (Z=-2.9, p=0.004).

S4. Relationship of atrophy-based typicality and severity with white matter hyperintensity

**Subtype dimension**	**Group**	**White matter hyperintensity**
Typicality	Aβ+	r_{part}=0.0007<br>p=0.99
	Aβ−	r_{part}=-0.11<br>p=0.18
Severity	Aβ+	r_{part}=-0.03<br>p=0.75
	Aβ−	r_{part}=.0.10<br>p=0.19

Linear partial correlation models examining relationship between typicality/severity with white matter hyperintensity. Each model was controlled for global Aβ SUVR, global tau SUVR and age. Additionally, models for typicality were controlled for severity and vice versa. Abbreviations: Aβ=amyloid-beta; SUVR= Standardized Uptake Value Ratio; r_{part}=linear partial correlation coefficient.

S5. Relationship of atrophy-based typicality and severity with cognitive performance controlled for Aβ, tau and white matter hyperintensity burden

Subtype dimension	Group	Memory	Executive function	Language	Visuospatial
Typicality	Aβ+	**r_{part}=0.49 p<0.01**	**r_{part}=0.17 p=0.04**	**r_{part}=0.19 p=0.02**	r_{part}=-0.01 p=0.95
	Aβ−	r_{part}=0.04 p=0.61	r_{part}=-0.03 p=0.67	r_{part}=-0.12 p=0.12	r_{part}=0.05 p=0.68
Severity	Aβ+	**r_{part}=0.26 p<0.02**	**r_{part}=0.25 p=0.002**	**r_{part}=0.29 p<0.01**	r_{part}=0.12 p=0.28
	Aβ−	r_{part}=0.02 p=0.76	r_{part}=-0.06 p=0.43	r_{part}=0.05 p=0.50	r_{part}=-0.16 p=0.21

Linear partial correlation models examining relationship between typicality/severity with cognitive performance. All models were controlled for pathological burden (global Aβ SUVR, global tau SUVR, estimated intracranial volume adjusted white matter hyperintensity volume). Models for typicality were controlled for severity and vice versa. Abbreviations: Aβ=amyloid-beta; r_{part}=linear partial correlation coefficient.

S6. Pathological contributors (Aβ, tau and white matter hyperintensity) and atrophy-based (A) typicality and (B) severity as correlates of cognitive performance in the AD continuum

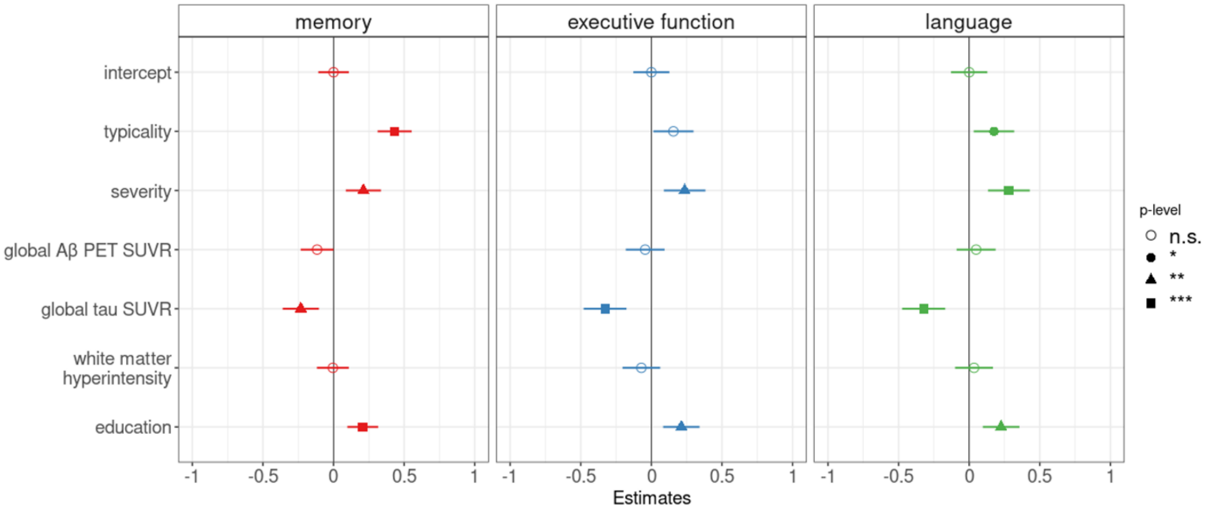

Follow-up analyses to significant associations observed in **S5**. Forest plot for multiple regression analyses showing the relative contribution of Aβ (global Aβ PET SUVR), tau (global tau PET SUVR) and cerebrovascular (estimated intracranial volume-adjusted white matter hyperintensity) pathologies in addition to typicality and severity to explain composite memory, executive function, and language scores. As potential covariates, age and education were tested in all models. As age appeared to be a non-significant contributor in all models, the variable was dropped from the final models. The vertical line indicates no effect. Standardized point estimates and error bars are shown, significance levels were corrected for multiple comparisons and correspond to $p<0.05$(*), $p<0.01$ (**) and $p<0.001$ (***).

S7. Sensitivity analysis to assess potential outlier effect

One individual was suspected to be an outlier in the AD continuum with relatively higher value of severity (total gray matter volume adjusted for estimated intracranial volume) observable in **Figure 1A** and **Figure 2** of the main text. This individual was 59 years old, male and *APOE ε4* carrier. Thus, we conducted sensitivity analysis by excluding this individual. Upon exclusion of this individual, the association between typicality and severity remained significant in the AD continuum (r = 0.34, p < 0.0001). We assessed the association between atrophy-based typicality/severity and pathological burden (global Aβ SUVR, global tau SUVR, estimated intracranial volume adjusted white matter hypointensity volume and adjusted for age). Consistent with the main results (**Table 2**), typicality (r_{part}=-0.31, p=0.002) and severity (r_{part}=-0.37, p<0.001) were associated with global tau SUVR. Further, we examined the association between atrophy-based typicality/severity and cognitive performance. The results were in alignment with the main results (**Table 3**): both typicality and severity were significantly associated with cognitive performance (memory, executive function, language domains) in the AD continuum after controlling for pathological burden. Specifically, typicality was significantly associated with memory (r_{part}=0.49, p<0.0001) and language (r_{part}=0.19, p=0.02) composite scores. Severity was significantly associated with memory (r_{part}=0.28, p=0.0006), executive function (r_{part}=0.25, p=0.002) and language (r_{part}=0.28, p=0.0006) composite scores. Finally, we evaluated the relative pathological variables contributing to cognitive performance beyond typicality and severity with multiple linear regression models for memory, executive function and language domains. Significant effects observed in the main findings (**Figure 3**) were also significant after excluding the potential outlier.

References

DeCarli, C, B L Miller, G E Swan, T Reed, P A Wolf, J Garner, L Jack, and D Carmelli. 1999. "Predictors of Brain Morphology for the Men of the NHLBI Twin Study." *Stroke* 30 (3): 529–36.

Voevodskaya, Olga, Andrew Simmons, Richard Nordenskjöld, Joel Kullberg, Håkan Ahlström, Lars Lind, Lars-Olof Wahlund, Elna-Marie Larsson, Eric Westman, and Alzheimer's Disease Neuroimaging Initiative. 2014. "The Effects of Intracranial Volume Adjustment Approaches on Multiple Regional MRI Volumes in Healthy Aging and Alzheimer's Disease." *Frontiers in Aging Neuroscience* 6: 264.
